# Autoplastic Treatment of Severe Burns

**Published:** 1870-09

**Authors:** 


### Autoplastic Treatment of Severe Burns.

In the service of Prof. Gosselin, at the Hospital of the Charity, has been, for the last six months, a man about forty years of age, who had the whole of his right leg severely burned about two years ago, for which, at that time he entered a hospital, and remained for six months. There being left a wound, about five inches long and one wide, which would not heal, he was then sent to Vincennes, where there is a hospital for men, convalescent, from all the hospitals of Paris. Having remained there a month or six weeks, and feeling strong, he resolved to leave the hospital and begin work. The little wound, still remaining open, now began to enlarge, and finally became so troublesome that the patient sought aid from Prof. Gosselin. This celebrated surgeon, from the beginning of last October till the middle of March, tried everything possible to make the wound heal. He finally gave up in despair, telling the patient he must leave the hospital, as he could do nothing for him. One of the internes of the service then proposed to try the autoplastic method, but in a new way. This latter consists in taking, with a lancet, little pieces of the patient's skin, or of another person's, and putting them on to the wound, where they soon begin to granulate, and around them the skin begins to grow, forming, as it were, little islands of true skin in the midst of the wound. The pieces of skin taken are not larger than the heads of two or three pins taken together, and just thick enough to cause a little bleeding of the part from which they are taken. I examined this patient's leg this morning, and found that the large exposed surface is nearly covered with true skin; and in a very short time he will be completely well, and out of the service. The patient said he felt nothing in the wound. All goes perfectly well.

In doing this little operation, which is certainly much to be recommended, care must be taken that the cut surface of the skin is placed on the surface of the wound, and there maintained with a strip of diachylum till it has formed a union with the part. The ordinary dressing may be placed over the adhesive strip of plaster and rest of the wound.

The interne, who appears to be the originator of this process, informed me that he had healed, in this manner, several wounds which had been considered hopeless. Prof. Gosselin has given him three or four patients in his private practice to treat, on whom he had been long uselessly trying to heal their wounds.

I saw, two days ago, Prof. Richet, ordering the same treatment for one of these indefinite wounds, result of a burn. He, also, has been trying for months to make the wound heal, and at last gave it up. Being told of this new treatment, he resolved to try it.

It is almost needless to state that, before applying these little pieces of borrowed skin, the wound should first be brought into as healthy a condition as possible; that it would be useless to apply them while the suppuration is very profuse.—*Paris Cor. Chicago Med. Jour.—N. Y. Med. Jour.*

